# Comparative salivary gland transcriptomics of sandfly vectors of visceral leishmaniasis

**DOI:** 10.1186/1471-2164-7-52

**Published:** 2006-03-15

**Authors:** Jennifer M Anderson, Fabiano Oliveira, Shaden Kamhawi, Ben J Mans, David Reynoso, Amy E Seitz, Phillip Lawyer, Mark Garfield, MyVan Pham, Jesus G Valenzuela

**Affiliations:** 1 Vector Molecular Biology Unit, Laboratory of Malaria and Vector Research, NIAID, NIH; 2 Centro de pesquisa Goncalo Moniz, Fundacao OswaldoCruz, and Faculdade de Medicina, Universidade Federal da Bahia, Salvador, Bahia, Brazil; 3 Laboratory of Parasitic Diseases, NIAID, NIH, Bethesda, MD, USA; 4 Vector Biology Section, LMVR, NIAID, NIH, Rockville, MD, USA; 5 Research Technologies Branch, NIAID, Rockville, MD, USA

## Abstract

**Background:**

Immune responses to sandfly saliva have been shown to protect animals against *Leishmania *infection. Yet very little is known about the molecular characteristics of salivary proteins from different sandflies, particularly from vectors transmitting visceral leishmaniasis, the fatal form of the disease. Further knowledge of the repertoire of these salivary proteins will give us insights into the molecular evolution of these proteins and will help us select relevant antigens for the development of a vector based anti-*Leishmania *vaccine.

**Results:**

Two salivary gland cDNA libraries from female sandflies *Phlebotomus argentipes *and *P. perniciosus *were constructed, sequenced and proteomic analysis of the salivary proteins was performed. The majority of the sequenced transcripts from the two cDNA libraries coded for secreted proteins. In this analysis we identified transcripts coding for protein families not previously described in sandflies. A comparative sandfly salivary transcriptome analysis was performed by using these two cDNA libraries and two other sandfly salivary gland cDNA libraries from *P. ariasi *and *Lutzomyia longipalpis*, also vectors of visceral leishmaniasis. Full-length secreted proteins from each sandfly library were compared using a stand-alone version of BLAST, creating formatted protein databases of each sandfly library. Related groups of proteins from each sandfly species were combined into defined families of proteins. With this comparison, we identified families of salivary proteins common among all of the sandflies studied, proteins to be genus specific and proteins that appear to be species specific. The common proteins included apyrase, yellow-related protein, antigen-5, PpSP15 and PpSP32-related protein, a 33-kDa protein, D7-related protein, a 39- and a 16.1- kDa protein and an endonuclease-like protein. Some of these families contained multiple members, including PPSP15-like, yellow proteins and D7-related proteins suggesting gene expansion in these proteins.

**Conclusion:**

This comprehensive analysis allows us the identification of genus- specific proteins, species-specific proteins and, more importantly, proteins common among these different sandflies. These results give us insights into the repertoire of salivary proteins that are potential candidates for a vector-based vaccine.

## Background

Phlebotomine sandflies are vectors of *Leishmania *parasites, causal agents of leishmaniasis in at least 88 countries. The manifestations of this disease range from the self-healing cutaneous and mucocutaneous forms to the potentially fatal visceral form. The incidence of leishmaniasis is 2 million cases annually, of which 500,000 cases are visceral and potentially lethal [1]. Visceral leishmaniasis is caused by parasites of the *Leishmania donovani *complex: *L. donovani, L. infantum and L. chagasi *(*L. infantum chagasi*). There are a limited number of competent sandfly vectors that can transmit parasites within this complex. For example, *Phlebotomus argentipes *transmits only *L. donovani *in the India sub-continent, *P. ariasi *and *P. perniciosus *transmit *L. infantum *within southern Europe, and *Lutzomyia longipalpis *exclusively transmits *L. chagasi *(*L. infantum chagasi*) in Central and South America.

Infected sandflies deliver the *Leishmania *parasite to a mammalian host during blood meal acquisition. Together with the parasite, sandflies inject saliva into the host. This saliva contains potent pharmacologically active components that facilitate blood feeding [2]. Additionally, the saliva affects the establishment of the parasite within the vertebrate host; small amount of *L. longipalpis *saliva exacerbates *L. major *infection in mice [3]. On the other hand, immune responses to sandfly saliva have been shown to protect against *Leishmania *infection [4,5]. Antibodies to maxadilan, a salivary protein from the sandfly *L. longipalpis *protected mice against *L. major *infection [6], while cellular immune response to PpSP15, a protein from the sandfly *P. papatasi *was sufficient to control *L. major *infection in mice [7]. Therefore, immune responses to salivary protein have promise as an effective vector-based vaccine to control *Leishmania *infection.

The repertoire of sandfly salivary proteins that have been studied is largely limited to three sandflies: *P. papatasi, P. ariasi *and *L. longipalpis*, vectors of *L major, L. infantum *and *L. chagasi (L. infantum chagasi)*, respectively. Only two salivary proteins have been extensively studied: maxadilan from the sandfly *L. longipalpis*, and PpSP15 from the sandfly *P. papatasi*. Maxadilan, a potent vasodilatory, immunomodulatory and protective molecule was shown to be very polymorphic [8]. On the other hand, PpSP15, a protective molecule with unknown biological function was shown to be highly conserved among colonized or field-collected *P. papatasi *sandflies [9].

Because of the potential of sandfly salivary proteins as anti-*Leishmania *vaccines, it is important to understand the diversity and degree of similarity between salivary proteins from various sandflies. More importantly, understanding the evolutionary relatedness of salivary proteins will help us to identify proteins that can be used as a global or general anti-*Leishmania *vaccine within a complex of vector species.

Here we explored the proteins and transcripts encoded in the salivary glands of the sandflies *P. argentipes *and *P. perniciosus *and studied the repertoire of proteins on these sandflies and compared them with the *P. ariasi and L. longipalpis *salivary proteins, also vectors of visceral leishmaniasis. We studied their molecular characteristics in the relation to molecular evolution of sandfly salivary proteins, and in the context of possible scenarios for global vector-based vaccines strategies.

## Results and discussion

### Sequencing of *P. argentipes *and *P. perniciosus *salivary gland cDNA libraries

From the *P. argentipes *salivary gland cDNA library, we sequenced 603 randomly selected clones from which 135 unique clusters of related sequences were obtained. Out of the 135 clusters, we found 45 clusters (1.11 sequences per cluster) of transcripts coding for housekeeping genes. We found 111 sequences, arranged in 55 clusters (1.8 sequences per cluster) that were not similar to other genes in the NCBI databank and lacked a secretory signal peptide. The most abundant transcripts in this cDNA library contained putative secretory proteins. We found 438 cDNA with potentially secreted proteins signals arranged in 30 clusters (an average of 14.36 sequences per cluster). The number of cDNA coding for secretory proteins is 9 times greater than the cDNA coding for housekeeping genes and 4 times greater than the cDNA coding for non-secreted proteins with unknown function. The transcripts coding for secretory proteins represent 73% of the total transcripts sequenced in the *P. argentipes *salivary gland library.

Similarly, the most abundant transcripts found in the *P. perniciosus *salivary gland cDNA library coded for secretory proteins. From a total of 535 sequenced cDNA we found that 394 cDNA were potentially secreted and were grouped into 30 clusters (average of 14.36 sequences per cluster). The cDNA coding for secretory proteins represent 74% of the cDNA sequenced, while transcripts coding for housekeeping genes represent 13.4 % (72/535). An additional 13 % (69/535) of transcripts coded for unknown (non-secreted) proteins. Table 1 and Table 2 contain the results of the analysis of the transcripts coding for secreted proteins from the salivary glands of *P. argentipes *and *P. perniciosus*.

**Table 1:** Putative secreted proteins from the salivary glands of *Phlebotomus argentipes*.

**Sequence name**	**NCBI accession number**	**Cluster**	**Signal P site**	**MW**	**pI**	**Best match to NR protein database**	**E value**	**Comments**	**Present in proteome**
PagSP01	DQ136148	1	20–21	14.2	6.1	SL1 protein *L. longipalpis*	2e-018	Similar to PpSP15	Yes
PagSP02	DQ136149	2	20–21	13.6	9.4	SL1 protein *L. longipalpis*	7e-029	Similar to PpsP15	Yes
PagSP03	DQ136150	3	21–22	34.9	9.1	Apyrase *Phlebotomus*	4e-092	Salivary apyrase	Yes
PagSP04	DQ136151	4	18–19	43.2	8.8	44 kDa salivary protein	1e-120	Yellow protein	Yes
PagSP05	DQ136152	5	19–20	29.1	9.1	Antigen 5 *L. longipalpis*	1e-104	Antigen 5 protein	Yes
PagSP06	DQ136153	6	17–18	24.9	9.6	32 kDa salivary protein	9e-038	Similar to PpSP32	Yes
PagSP07	DQ136154	7	20–21	14.3	8.9	SL1 protein *L. longipalpis*	2e-019	Similar to PpSP15	Yes
PagSP09	DQ136155	9	22–23	33.1	8.9	32 kDa protein *L. longipalpis*	2e-64		Yes
PagSP10	DQ136156	10	19–20	26.7	5.5	28 kDa salivary protein	7e-078	D7 related protein	Yes
PagSP11	DQ136157	11	21–22	40.1	9.4	Endonuclease *L. longipalpis*	4e-039	Endonuclease	
PagSP12	DQ136158	12	20–21	14.1	8.9	SL1 protein *L. longipalpis*	4e-023	Similar to PpSP15	
PagSP13	DQ136159	13	20–21	13.9	8.9	14 kDa salivary protein	8e-031	Similar to PpSP15	
PagSP14	DQ136160	14	24–25	43.9	8.8	ebiP3881 *An gambiae*	7e-099	Lipase-like	
PagSP15	DQ136161	15	20–21	30.1	9.6			Novel protein	
PagSP17	DQ136162	17	20–21	29.4	8.0			Novel protein	Yes
PagSP19	DQ136163	19	21–22	30.0	12			Novel protein	
PagSP20	DQ136164	20	20–21	27.4	9.6			Novel protein	
PagSP25	DQ136165	25	20–21	27.9	9.5	30 kDa salivary protein	6e-045	D7 protein	
PagSP56	DQ136166	56	20–21	30.1	7.0			Novel protein	
PagSP60	DQ136167	60	19–20	11.7	3.9	putative histone promoter	2e-005		
PagSP73	DQ136168	73	20–21	16.1	5.5	*A. gambiae *unknown	0.002	Unknown	
PagSP124	DQ136169	124	21–22	15.5	5.2			Novel protein	
PagSP132	DQ136170	132	22–23	47.3	6.5	agCP4255 *An. gambiae*	2e-095	Pyrophosphatase	

**Table 2:** Putative secreted proteins from the salivary glands of *Phlebotomus perniciosus*.

**Sequence name**	**NCBI accession number**	**Cluster**	**Signal P site**	**MW**	**pI**	**Best match to NR protein database**	**E value**	**Comments**	**Present in proteome**
PpeSP01	DQ192490	1	20–21	35.5	9.3	Salivary apyrase *P. papatasi*	4e-86	Apyrase	Yes
PpeSP01B	DQ192491	1B				Salivary apyrase *P. papatasi*		Apyrase	Yes
PpeSP02	DQ150620	2	20–21	14.8	8.7	SL1 protein *L. longipalpis*	2e-20	SP15 like protein	Yes
PpeSP03	DQ150621	3	18–19	41.8	6.0	42 kDa salivary prot. *P. papatasi*	1e-113	Yellow protein	Yes
PpeSP03B	DQ150622	3B	18–19	42.7	8.6	44 kDa salivary prot. *P. papatasi*	1e-117	Yellow protein	Yes
PpeSP04	DQ150623	4	19–20	24.5	8.5	28 kDa salivary prot. *P. papatasi*	6e-64	D7 protein	Yes
PpeSP04B	DQ150624	4B	19–20	26.9	8.7	28 kDa salivary prot. *P. papatasi*	3e-80	D7 protein	Yes
PpeSP05	DQ153099	5	17–18	27.8	10.4	29 kDa salivary prot. *L. longipalpis*	1e-23		
PpeSP06	DQ153100	6	22–23	33.0	8.9	32 kDa salivary prot. *L. longipalpis*	6e69		Yes
PpeSP07	DQ153101	7	19–20	29.6	9.1	Antigen 5 prot. *L. longipalpis*	5e-84	Antigen 5 protein	Yes
PpeSP08	DQ153102	8	25–26	28.8	4.9	Salivary prot. *C. sonorensis*	0.016		
PpeSP09	DQ153103	9	20–21	14.6	8.6	14 kDa salivary prot. *P. papatasi*	2e-28	SP15-like protein	Yes
PpeSP10	DQ153104	10	19–20	26.7	9.4	30 kDa salivary prot. *P. papatasi*	2e-47	D7 protein	Yes
PpeSP11	DQ153105	11	19–20	13.2	9.0	SL1 prot. *L. longipalpis*	1e-20	SP15 like protein	Yes
PpeSP12	DQ153106	12	20–21	7.1	11.0			Novel protein	
PpeSP13	DQ153107	13	20–21	9.7	4.8			Novel protein	
PpeSP15	DQ192489	15	25–26	2.7	10.6			Novel protein	
PpeSP18	DQ154097	18	29–30	29.9	8.3	Phospholipase A2, *Drosophila*	1e-78	Phospholipase A2	
PpeSP19	DQ154098	19	20–21	45.8	8.5	37 kDa prot.. *L. longipalpis*	2e-33		Yes
PpeSP32	DQ154099	32	23–24	41.4	9.5	*L. longipalpis *endonuclease	1e-121	Endonuclease	

In addition to the identification of proteins previously reported from other sandflies, we found a number of transcripts coding for proteins not previously shown to be present in the salivary glands of sandflies. A protein homologous to lipases from *Anopheles gambiae*, *Drosophila melanogaster *and other organisms was found in the *P. argentipes *cDNA library. We also found in this library and in the *P. perniciosus *cDNA library, a transcript coding for a protein homologous to a pyrophosphatase. The predicted 47-kDa protein named PagSP132 contains a phosphodiesterase type I, phosphodiesterase/nucleotide pyrophosphatase motif. This type of enzymes cleaves the phosphodiester and phosphosulfate bonds in NAD, deoxynucleotides and nucleotide sugars [10]. BLAST search of this protein identified protein orthologs found in mammals as well as in *A. gambiae*. To our knowledge, pyrophosphatases have not been described in the saliva of other sandflies.

We found one cluster in the *P. perniciosus *cDNA library coding for a phospholipase A2 (PLA2) protein (PpeSP18). This type of protein has never been reported from the saliva of a blood-feeding insect. PLA2 (Phosphatidylcholine-2-acylhydrolase, E.C. 3.1.1.4) are well known for their ability to cleave the arachidonic acid and lysophosphatidylcholine from the sn-2 position of membrane glycerol-3-phospholipids. Also PLA2 are known to work as toxins by blocking the release of neurotransmitters [11].

We identified transcripts coding for secreted proteins that did not match any reported proteins in accessible databases. *P. argentipes *contained six unknown proteins that ranged from 15 to 30 kDa, while only three were found in the *P. perniciosus *library and all were relatively small ranging from 10 to 27 kDa (Tables 1 and 2).

### Proteome analysis of *P. argentipes *and *P. perniciosus *salivary proteins

**Figure 1:**
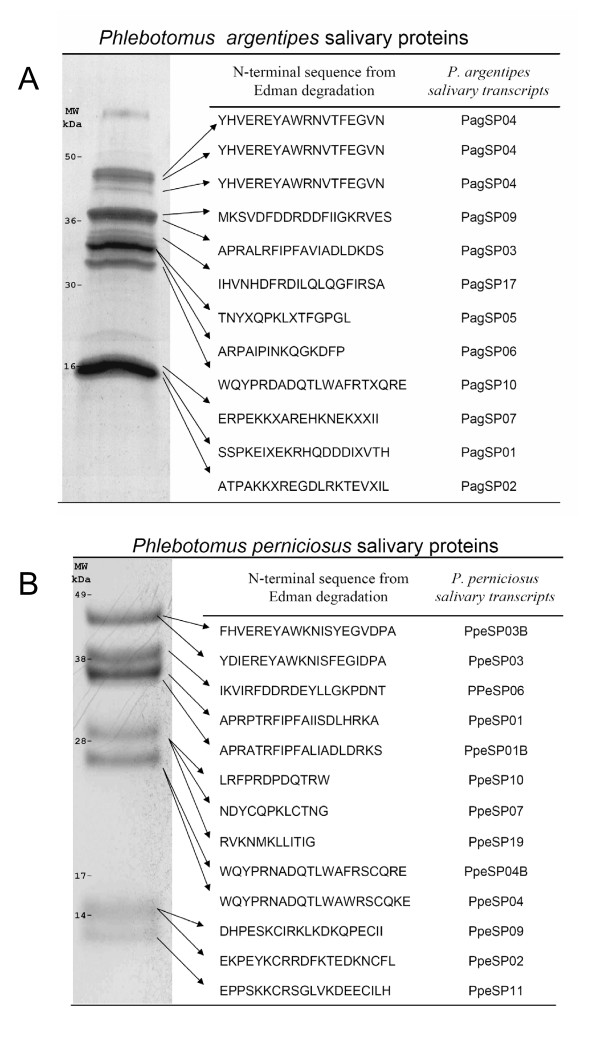
**Amino-terminal sequence of salivary gland proteins**. (A) Supernatant of salivary gland homogenate of *Phlebotomus argentipes *was separated in SDS-PAGE and transfer to PVDF membrane as described in Methods. N-terminal sequence obtained was searched in the sandfly database and the clone containing the sequence is shown at the right. (B) N-terminal sequence and matched clones of salivary gland proteins from *Phlebotomus perniciosus*.

Edman degradation of the salivary proteins separated by SDS-PAGE from *P. argentipes *resulted in the identification of 12 N-terminal sequences (Figure 1A). The identified proteins included three PpSP15-like protein (PagSP02, PagSP01 and PagSP07), D7-related protein (PagSP10), PpSP32-like salivary protein (PagSP06), antigen 5 related proteins (PagSP05), a novel protein (PagSP17), *P. papatasi *apyrase-like protein (PagSP03), *L. longipalpis *32-kDa-like salivary protein (PagSP09), and a yellow-related protein (PagSP04). Three proteins with different mobility on the gel had the same N-terminal sequence (PagSP04), and were probably derived from the same transcript but with different post-translational modifications.

From *P. perniciosus *salivary gland protein analysis we found 13 N-terminus sequences (Figure 1B). The identified proteins included: three PpSP15-like proteins (PpeSP11, PpeSP02 and PpeSP09), three D7-related proteins (PpeSP04, PpeSP04B, PpeSP10), a 37-kDa-like protein described previously in the saliva of *L. longipalpis *(PpeSP19), an antigen-5 related protein (PpeSP07), two apyrase-like proteins (PpeSP01, PpeSP01B), a 32-kDa-like salivary protein described on *L. longipalpis *(PpeSP06) and two yellow-related proteins (PpeSP03, PpeSP03B). Not all attempted Edman degradation experiments resulted in a sequence, either because of insufficient protein amount or because N-terminal ends were blocked.

### Comparative analysis of salivary transcripts from vectors of visceral leishmaniasis: *P. argentipes, P. perniciosus, P. ariasi and L. longipalpis*

In an attempt to understand the relationship of salivary proteins among different sandflies and to gain insights into the evolution of sandfly salivary proteins, we compared cDNA libraries from four different sandflies. We selected the sandflies based on their availability and their significance for this study. First, we selected sandflies from the two different genera, *Phlebotomus *and *Lutzomyia*. Secondly, from the *Phlebotomus *genus we selected two different subgenus, *Euphlebotomus *(*P. argentipes*) and *Larroussius *(*P. ariasi *and *P. perniciosus*). The phylogenetic relationship among these sandflies was previously studied using the small subunit nuclear ribosomal DNA [12].

Full-length secreted proteins from each sandfly library were compared using a stand alone version of BLAST. We found 10 families of proteins that are common among all four cDNA libraries: 1) PpSP15 like protein, 2) apyrase-like, 3) yellow related protein, 4) antigen-5 related protein, 5) PpSP32 like protein, 6) 32 kDa-like protein, 7) D7 related protein and 8) an endonuclease-like protein, 9) a 39-kDa-like protein, and a 16.1 kDa-like protein (Table 3). The level of similarities according to BLAST values was highly significant (2E^-18 ^to 1E^-169^). The protein families listed above may be common to both *Lutzomyia *and *Phlebotomus *species, and may be present in other species from both genera.

**Table 3:** Salivary transcripts shared by *Phlebotomus *and *Lutzomyia *sandflies.

**Family of proteins**	***P. argentipes***	***P. ariasi***	***P. perniciosus***	***L. longipalpis***
**PpSP15-like protein**	PagSP01, 02, 07, 12, 13	ParSP03, 08	PpeSP02, 09, 11	LJM04
**Apyrase**	PagSP03	ParSP01	PpeSP01, 01B	LJL23
**Yellow protein**	PagSP04	ParSP04, 04B	PpeSP03, 03B	LJM17, LJM11, LJM111
**Antigen 5-related protein**	PagSP05	ParSP05	PpeSP07	LJL34
**PpSP32-like protein**	PagSP06	ParSP02	PpeSp05	LJL04
**33 kDa, unknown function**	PagSP09	ParSP09	PpeSP06	LJL143
**D7-related protein**	PagSP10, 25	ParSP07, 12, 16	PpeSP04, 04B, 10	LJL13
**Endonuclease-like**	PagSP11	ParSP10	PpeSP32	LJL138
**39 kDa, unknown function**		ParSp17	PpeSP19	LJM78
**16.1 kDa, unknown function.**		ParSP80		LJS138

It is interesting to note the amount of variation that exists in the number of members of the different protein families found in the four sandflies (Table 3). The PpSP15-like family has five members identified in *P. argentipes *(PagSP01, PagSP02, PagSP07, PagSP12 and PagSP13), two in *P. ariasi *(ParSP03, ParSP08), three in *P. perniciosus *(PpeSP02, PpeSP09 and PpeSP11), yet only one (LloSP05) in *L. longipalpis *(Table 3). On the other hand, only one member of the apyrase family of proteins has been found in each sandfly except for *P. perniciosus*, which has two members (Table 3). Other families of salivary proteins, such as antigen 5, PpSP32, 32 kDa and endonuclease-like protein were represented by only one member from each of the different sandflies. The yellow-related protein has one member found in *P. argentipes*, two members in the *P. perniciosus *and *P. ariasi *and three members in the *Lutzomyia longipalpis *sandfly. The D7-related protein was represented by three members in *Phlebotomus *sandflies, while only one member is present in the *L. longipalpis *sandfly.

Through comparative analysis we found at least one unique transcript from each of the four cDNA libraries. Nine unique transcripts were identified in *P. argentipes*, five in *P. ariasi*, one in *P. perniciosus *and twenty-four in *L. longipalpis*. The large difference in *L. longipalpis *may be due to genus differences (*Phlebotomus *vs. *Lutzomyia*); therefore, we would expect to find similar transcripts in other *Lutzomyia *species. Only four families of proteins were unique to *Phlebotomus*: a 32-kDa protein of unknown function, a 2-kDa peptide, a 5-kDa peptide and a phospholipase A2-like protein (Table 4).

**Table 4:** Salivary transcripts shared by *Phlebotomus *sandflies.

**Protein**	***P. argentipes***	***P. ariasi***	***P. perniciosus***
**32 kDa, unknown function**	PagSP19	ParSP25	PpeSP08
**2 kDa, unknown function**		ParSP23	PpeSP15
**5 kDa, unknown function**		ParSP15	PpeSP12
**Phospholipase A2-like**	PagSP18	ParSP11	PpeSP18

### Molecular characteristics of salivary proteins shared among the analysed sandflies

In order to understand the relationship among salivary proteins from different sandflies, we performed multiple sequence alignment followed by phylogenetic analysis of the salivary transcripts shared by the vectors of visceral leishmaniasis studied (Table 3). Following is a description of the shared proteins:

#### SL1/PpSP15 related proteins

The SL1/PpSP15 group of proteins is similar to the SL1 salivary protein from *L. longipalpis*, which has no known function [13], and to PpSP15, a 15- kDa salivary protein from *Phlebotomus papatasi *that was previously shown to confer protection against *L. major *infection [14]. The predicted molecular weight of these transcripts is approximately 14 kDa and is in agreement with the observed MW found through the proteome analysis (Figure 1). This group represents the most abundant transcripts in the salivary gland cDNA library of *P. argentipes *(Table 1). The fact that only one PpSP15 member was found in *L. longipalpis*, suggests that a number of lineage-specific gene expansions (gene duplication events) occurred in the *Phlebotomus *lineage at various periods in the evolution of these sandflies.

The PpSP15 family of proteins has only been found in species of sandflies suggesting that this family was a specific invention that occurred during sandfly evolution, most likely during their adaptation to a blood-feeding environment. Although PSI-BLAST analysis using PpSP15 proteins retrieved only members of the PpSP15 family, the PHYRE prediction servers indicated that members of the PpSP15 family possess an EF-hand fold most closely related to members of the odorant-binding protein (OBP) family to which the D7-proteins belong. It is thus likely that PpSP15 members were derived from an OBP ancestral protein. Characteristically, the OBP family in *Drosophila *has a low degree of sequence similarity among its members with only six conserved cysteines among the thirty-four members of this family [15].

**Figure 2:**
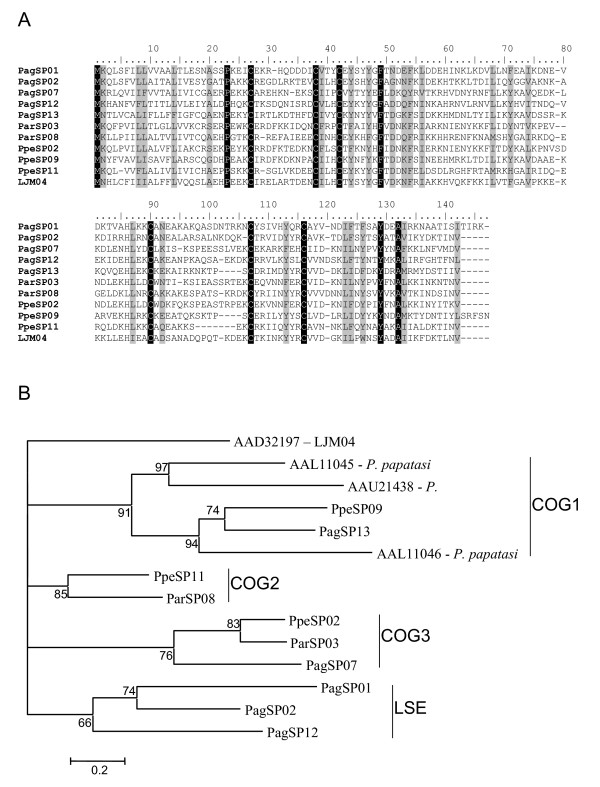
**Analysis of PpSP15 family of sandfly salivary proteins**. (A) Multiple sequence alignment of PpSP15 salivary proteins from *Phlebotomus argentipes *(Pag), *Phlebotomus ariasi *(Par), *Phlebotomus perniciosus *(Ppe) *and Lutzomyia longipalpis *(LJ). Sequences were aligned using ClustalX and manually refined using BioEdit sequence-editing software. (B) Phylogenetic tree analysis of PpSP15 salivary proteins from these four sandflies. Phylogenetic analysis was conducted on protein alignments using Tree Puzzle version 5.2 by maximum likelihood using quartet puzzling, automatically estimating internal branch node support (1000 replications).

The multiple pair-wise alignment analysis of sandfly PpSP15 shows a high degree of divergence among the sequences, 7.5% identity and 23.10% similarity (Figure 2A). The number of amino acids between the second and third cysteine (three) and between the fifth and the sixth cysteine (eight) were shown to be conserved among all the Drosophila OBP members [15]. All of the sandflies sequences analysed from the PpSP15 protein family contained identical cysteine positioning. This data suggests that this family of proteins may be closely related to the short form of D7 because of the similarities to OBP and its small size of 15 kDa, which is similar to the MW found in the mosquitoes short D7 [16].

Phylogenetic analysis of PpSP15 transcripts from the different sandflies including *P. papatasi *resulted in the formation of 5 distinct clades (Figure 2B). As such, three major clusters of orthologous groups of proteins (COGs), and hence gene duplication events, can be identified that possibly occurred in the ancestor to the *Phlebotomus *lineage. COG1 includes members from *P. perniciosus*, *P. argentipes *and *P. papatasi *with a second related clade that includes a lineage-specific expansion (LSE) in *P. papatasi*. COG2 includes members from *P. perniciosus *and *P. argentipes*. COG3 includes members from *P. perniciosus*, *P. argentipes *and *P. ariasi*. Another clade composed solely of members of *P. argentipes *indicates another LSE. Evolutionarily, the *P. papatasi *group was basal to the other *Phlebotomus *members analysed in this study, followed by *P. argentipes*, with *P. perniciosus *and *P. ariasi *forming the terminal clade. Given this, the topology of the cladogram obtained for the PpSP15 family suggests that COG2 resulted from gene duplication event that occurred in the ancestor to *P. perniciosus *and *P. ariasi*. COG1 follows the expected phylogenetic grouping but suggests that this gene was lost in *P. ariasi *or we failed to detect the ortholog in our library. COG3 again suggests that this specific gene duplication event occurred after divergence from the shared ancestor with *P. papatasi*. The PpSP15 family found in sandflies is thus characterised by both gene duplication and possibly gene loss events, both restricting an accurate reconstruction of its phylogeny. It is currently impossible to say which proteins share a conserved function with the PpSP15 from *L. longipalpis *as the major clades create a polytomy. As such, this family might bind related or similar pharmacologic components so that they all have, in fact, a similar function. This might explain the seemingly haphazard acquisition and loss of genes.

#### D7 family of proteins

D7-related proteins are found in the saliva of different diptera, including *Anopheles *[17], *Aedes *[18] and *Culex *[19] mosquitoes as well as in the sandflies *P. papatasi *[19], *L. longipalpis *[13] and *P. ariasi *[21] and does not appear to occur in non-dipteran species. Two forms of the protein have been described; a long and short form [19]; and appear to be distantly related to an odorant binding super family of proteins. Interestingly, the OBP family seems to be the ancestral molecule of the PpSP15 family (see above). Therefore, it may be possible that both D7 and PpSP15 related proteins have a common ancestor.

The D7 protein named hamadarin from *Anopheles stephensi *acts as an anticoagulant affecting the plasma contact system by inhibiting the activation of Factor XII and kallikrein [23]. Recently, a biological function of four short members of the D7 family from *A. gambiae *and a long D7 from *Aedes aegypti *was described [16]. These salivary proteins were shown to bind biogenic amines such as serotonin, histamine and norepinephrine. This function is relevant for blood-feeding because of the inhibition of the vasoconstrictor, platelet aggregating, and pain inducing properties of these biogenic amines [16]. The exact function of D7 proteins in sandflies is largely unknown, but it may be related to the function observed in mosquito D7 proteins, either as an anticoagulant or binding biogenic amines.

The D7 family is represented in the *P. argentipes *cDNA library by two members, PagSP10 and PagSP25, and in the *P. perniciosus *cDNA library by three members, PpeSP04, PpeSP04B and PpeSP10. Only one member of this family is present in *L. longipalpis *sandfly, suggesting a case of gene duplication of this protein that probably occurred more recently in the *Phlebotomus *genus.

**Figure 3:**
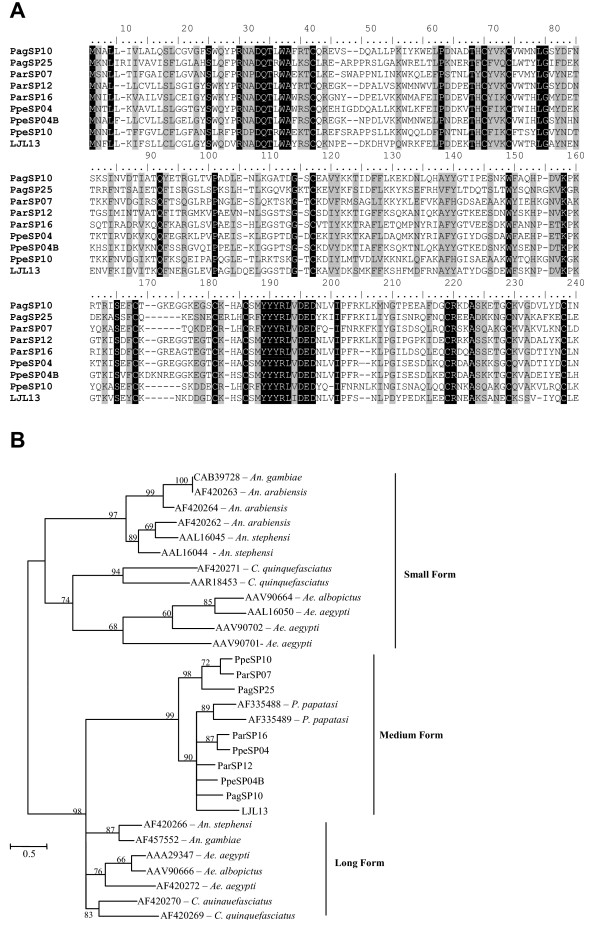
**Analysis of D7-related proteins**. A) Multiple sequence analysis of D7 proteins from the salivary glands of *Phlebotomus argentipes *(Pag), *Phlebotomus ariasi *(Par), *Phlebotomus perniciosus *(Ppe) *and Lutzomyia longipalpis *(LJ). Sequences were aligned using ClustalX and manually refined using BioEdit sequence-editing software. (B) Phylogenetic tree analysis of D7 salivary proteins from *P. argentipes *(Pag), *P. ariasi *(Par), *P. perniciosus *(Ppe), *L. longipalpis *(LJ), *P. papatasi*, *Aedes aegypti*, *Ae. albopictus*, *Anopheles gambiae, An. Arabiensis, An. Darlingi, An. Stephensi *and *Culex quinquefasciatus*. Phylogenetic analysis was conducted on protein alignments using Tree Puzzle version 5.2 by maximum likelihood using quartet puzzling, automatically estimating internal branch node support (1000 replications).

Comparative analysis of the D7 family of proteins from different sandflies reveals few conserved regions of identity, with only 16% identity and 23% similarity between the sandfly D7 proteins. There are 10 conserved cysteines throughout the molecule. The size of the sandfly D7 proteins is slightly smaller than the long D7 forms found in mosquitoes. Additionally, sandfly D7 is missing the last cysteine that is present at the carboxy terminal region of the mosquito long- and short-form D7 and, instead, have a cysteine present between conserved cysteines 8 and 19 [19]. Based on PHYRE prediction results, the long-form D7 proteins have 16 alpha helix domains, the short form have 8 alpha helix domains, while the sandfly form have 13 domains. All three forms are associated to OBP, as mentioned above. Interestingly, the sandfly D7 proteins are predicted to contain a beta-strand domain starting at the 7^th ^cysteine and characterised by a repeat of tyrosines at amino acid 188 (Figure 3A), replacing the alpha helix domain found in the mosquito D7 proteins at the same position. Due to these differences we are categorising the sandfly D7 as the medium form (Figure 3B).

Phylogenetic analysis of D7 proteins from different organisms shows 2 distinct clades with the sandfly proteins branching from the long form (Figure 3B). All of the sandfly D7 members are clustered within one clade, distinct from the long form members. The clade containing the mosquito D7 short-form proteins contains two clusters, one containing the *Anopheles *mosquitoes and the other containing the *Culex *and *Aedes *species. The sandfly clade subdivides into two distinct clades. The lower clade shows 5 distinct groups, two of these groups represent COGs of *P. perniciosus *and *P. ariasi*, and the group of *P. papatasi *seems to be a case of lineage expansion. The lower clade shows a COG that includes *P. argentipes*, *P. ariasi *and *P. perniciosus *proteins, PagSP25, ParSP07 and PpeSP10.

#### Apyrase family of proteins

Both the *P. perniciosus *and *P. argentipes *libraries contained transcripts homologous to the *Cimex *family of apyrases [24], a protein also present in the saliva of *P. papatasi *[7] and *L. longipalpis *[13]. Apyrases are enzymes that function as potent anti-platelet factors by destroying or hydrolysing the platelet activator ADP. An orthologue was found in humans and the recombinant protein was shown to hydrolyse a variety of nucleoside di- and triphosphates, preferentially UDP, followed by GDP, UTP, GTP, ADP, and ATP [25,26].

**Figure 4:**
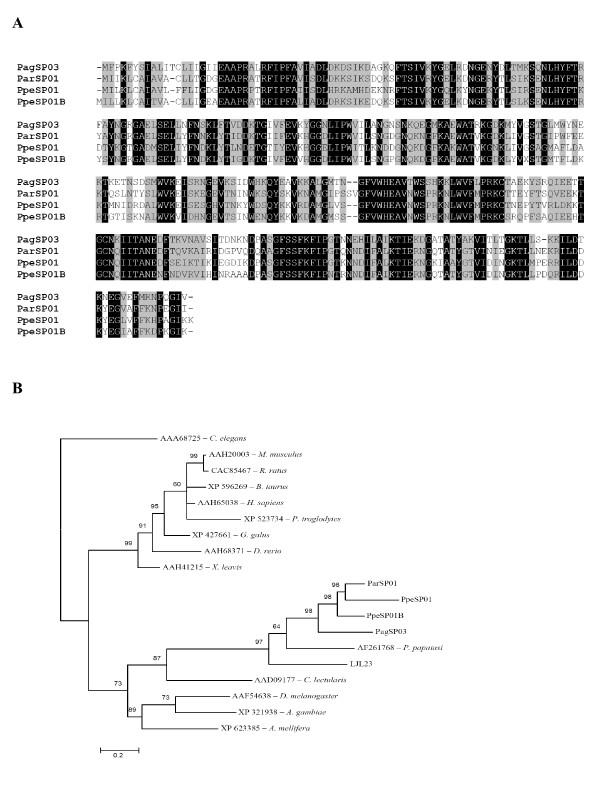
**Analysis of apyrase protein family**. (A) Multiple sequence analysis of apyrases from the salivary glands of *Phlebotomus argentipes *(Pag), *Phlebotomus ariasi *(Par) and *Phlebotomus perniciosus *(Ppe). Sequences were aligned using ClustalX and manually refined using BioEdit sequence-editing software. (B) Phylogenetic tree analysis of apyrase protein family from *P. argentipes *(Pag), *P. ariasi *(Par), *P. perniciosus *(Ppe), *L. longipalpis *(LJ), *P. papatasi*, *Cimex lectularius*, and transcripts coding from this protein family (identified at GenBank) from *Drosophila melanogaster, An. Gambiae, A. mellifera, C. elegans, M. musculus, R. ratus, B. Taurus, H. sapiens, P. troglodytes, G. galus, D. rerio *and *X. leavis*. Phylogenetic analysis was conducted on protein alignments using Tree Puzzle version 5.2 by maximum likelihood using quartet puzzling, automatically estimating internal branch node support (1000 replications).

Sequence alignment of the *P. argentipes*,*P. perniciosus *and *P. ariasi *apyrases show a 47% identity and 81% similarity at the amino acid level (Figure 4A). When *L. longipalpis *is included in the analysis, there is a considerable decrease in the identity (29%) as well as in the similarity (67%) (data not shown).

Phylogenetic analysis of apyrases from different organisms indicates three main clades with the sandfly apyrases in a distinct clade, apart from vertebrates, yet closely related to other insects (Figure 4B). Interestingly sandfly apyrases share a common ancestor with *Cimex lectularius *apyrase. The two insects appear to have evolved to the blood feeding mode independently [27]. Within the clade containing the sandflies, one of the *P. perniciosus *apyrases, PpeSP01, is more closely related to the *P. ariasi *apyrase (ParSP01) than the second apyrase from *P. perniciosus *(PpSP01B). This may be the result of a gene duplication event in *P. perniciosus *and subsequent loss in *P. ariasi*. When searching databases we found a transcript from *A. gambiae *similar to sandfly apyrases. This is interesting because the known mosquito apyrases belong to the 5'-nucleotidase family of proteins. The known mosquito apyrase is very distinct, in size and sequence, from the *Cimex *family of apyrases also present in sandflies [28,24,7], thus it is possible that mosquitoes in addition to a functional 5'-nucleotidase type apyrase, may have a non-functional sandfly/bedbug-like apyrase gene or it may have a house keeping function such as hydrolysing UDP formed after transglycosylation reactions in the Golgi [29]. Alternatively, the mosquito may have a similar apyrase but with different substrate specificity or this protein is not present in their salivary gland.

Interestingly, the mosquito apyrase gene seems to be ancestral to the sandfly apyrase based on phylogenetic association (Figure 4B). Then it may be possible that mosquitoes have lost the function of this gene and kept the active form of the 5'-nucleotidase gene, which is the active apyrase in these insects.

**Figure 5:**
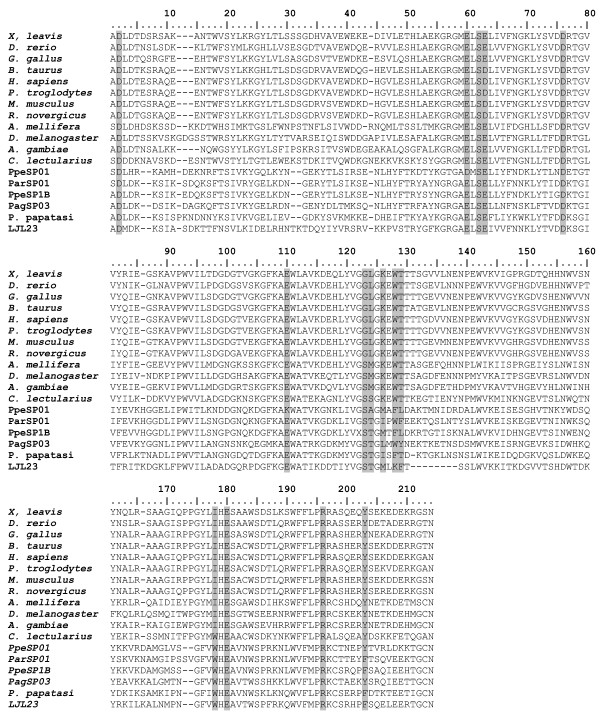
**Multiple sequence analysis of the apyrase family of proteins**. Multiple sequence alignment of different organisms showing the amino acids highlighted in grey that are relevant for calcium and nucleotide binding as predicted from the structure of the human apyrase. Sequences were aligned using ClustalX and manually refined using BioEdit sequence-editing software.

The crystal structure of the *Cimex *family of apyrases was elucidated from the human counterpart and the amino acids relevant for calcium- and nucleotide-binding sites were determined [30]. Several differences were noted among the amino acids relevant for nucleotide- or calcium-binding between sandflies, bedbugs and humans [30]. Figure 5 shows the alignment of the different apyrases with amino acids relevant for calcium- and nucleotide-binding highlighted. We observed clear differences in some of these amino acids when sandflies were compared with other organisms including mosquitoes. The amino acids at position 124 are Ser (S), Thr (T) or Ala (A), in sandflies and Met (M) or Leu (L) in other organisms (Figure 5). Amino acids at position 126 is Met (M), Ile (I) or Leu (L) in sandflies and Lys (K) in other organisms; at position 129, sandflies have either Lys (K), Tyr (Y) or Leu (L) and other organisms have only Thr (T). At position 178, sandflies and bedbugs have a Trp (W) while other organisms have Ile (I). Amino acid substitutions may produce the specificity of the sandfly apyrases to ADP, a molecule that these insects must hydrolyse to overcome the hemostatic system and take a successful blood meal. In fact, Dai et al [30] showed that amino acid substitutions at some positions changed the substrate (GDP to ADP) in human apyrase. Human apyrases have more affinity for GDP substrate while sandfly apyrases have affinity for ADP [30].

#### Yellow-related protein

The gene coding for the yellow protein was first described in *Drosophila melanogaster *[31]. The proteins in this family appear to be derived from a common ancestor of the major royal jelly proteins (MRJPs) from honeybees and the yellow protein from *Drosophila spp*.

*Drosophila *yellow protein is related to pigmentation and male sexual behavior. In the family Culicidae a yellow protein was identified in *Ae. aegypti *whole-larvae extract and was associated to a dopachrome converting enzyme activity found in this insect [32]. The function of this protein in the saliva of sandflies and its importance for blood feeding remains to be elucidated. The yellow protein family is one of the most abundant proteins found in the sandfly saliva.

We identified transcripts coding for secreted proteins of approximately 45 kDa, previously described in the saliva of *L. longipalpis*, *P. papatasi *and *P. ariasi *as yellow-related proteins [13,14,21]. In the *P. argentipes *cDNA library we found only one cluster (PagSP04) coding for this protein, yet in *P. papatasi *and *L. longipalpis *salivary glands there are multiple members of this family of proteins [33]. Proteomic analysis (Figure 1A) revealed that the PagSP04 transcript (YHVEREYAWRNVTFEGVN) was one of the most abundant proteins found in the salivary glands of *P. argentipes*. Interestingly, three proteins with different mobilities coded for the same N-terminus sequence (Figure 1A) suggesting they may represent the same protein with different post-translational modifications.

**Figure 6:**
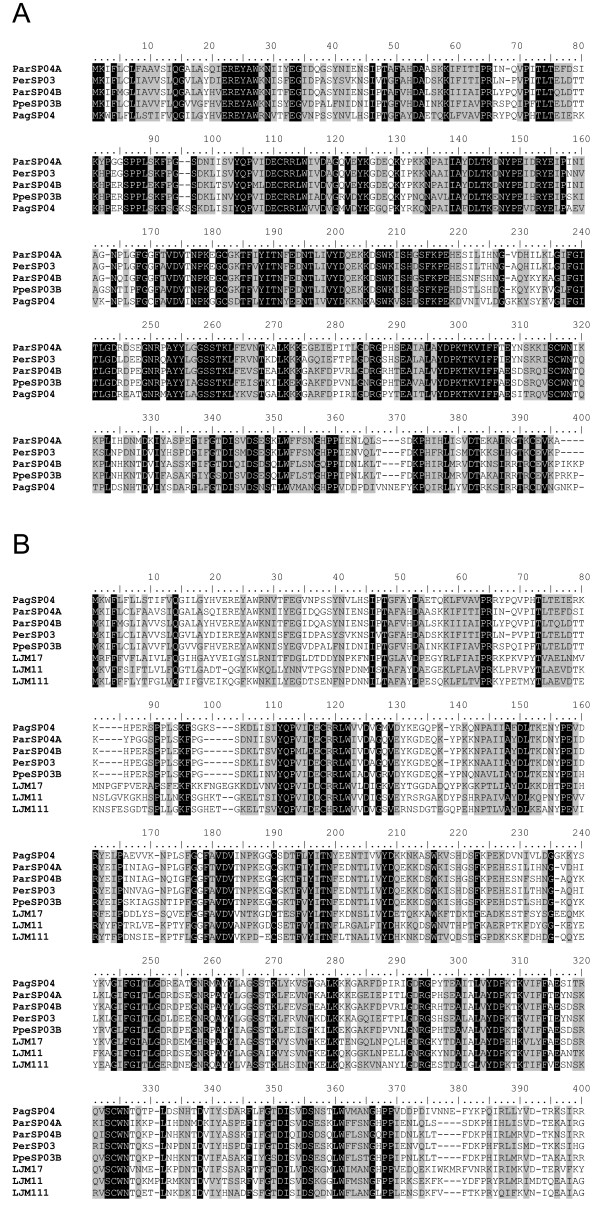
**Multiple sequence analysis of yellow-related proteins from the salivary glands of different sandflies**. (A) Only *Phlebotomus *species were compared. (B) A combination of *Phlebotomus *and *Lutzomyia *sandfly salivary yellow-related proteins is shown on this alignment. Sequences were aligned using ClustalX and manually refined using BioEdit sequence-editing software.

**Figure 7:**
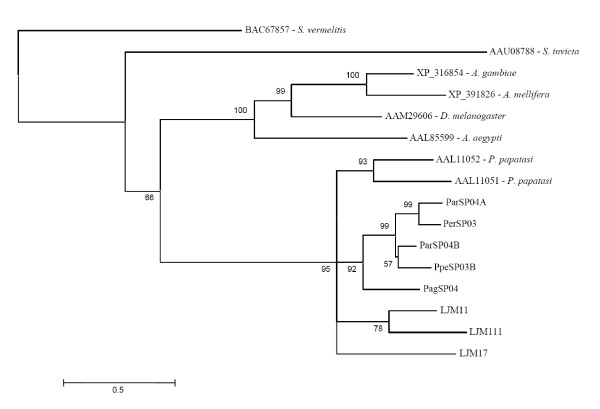
**Phylogenetic tree analysis of yellow-related salivary proteins**. Yellow-related proteins from other organisms including *S. invicta, An. Gambiae, A. mellifera, D. melanogaster *and *Ae. Aegypti*. Phylogenetic analysis was conducted on protein alignments using Tree Puzzle version 5.2 by maximum likelihood using quartet puzzling, automatically estimating internal branch node support (1000 replications).

Based on comparative analysis, we identified ten different sandfly salivary proteins that are members of the yellow family. Alignment of yellow proteins from *Phlebotomus *sandflies (*P. argentipes*, *P. perniciosus*, and *P. ariasi)*, revealed a 43% identity and 79% similarity among the members of this protein family (Figure 6A). When the three yellow proteins from *L. longipalpis *were added, the identity was 21% identity and similarity was 57% among these proteins (Figure 6B). The phylogenetic analysis based on maximum likelihood using amino acid data of several MRJP/yellow proteins resulted in the formation of various clades (Figure 7), one clade containing yellow proteins from honeybees, a second clade containing mosquitoes and *Drosophila*, and a third clade containing the yellow proteins from sandflies. The sandfly clade was subdivided into three sub-clades, one containing the two yellow proteins from *P. papatasi*, the second clade containing the yellow proteins from *P. ariasi *and *P. pernicious*, and the third clade containing the *L. longipalpis *yellow proteins.

Based on their MW, the yellow proteins from *L. longipalpis *appear to be the most recognised proteins from the sera of individuals living in endemic areas of visceral leishmaniasis and from individuals that have anti-*Leishmania *immunity [34]. The antibody response against these salivary proteins appears to correlate with protection against leishmaniasis.

#### Antigen-5 family of proteins

This cluster codes for a secreted protein of 29 kDa similar to antigen 5-related protein found in wasp venom [35]. Similar proteins have been isolated from the salivary glands of *Aedes aegypti *[20], *An. gambiae *[36] and from the salivary glands of *L. longipalpis *[13]. We found only one cluster coding for this protein in the cDNA library of *P. argentipes *(PagSP05) and in the cDNA library of *P. perniciosus *(PpeSP07). The N-terminal sequence corresponding to these transcripts was identified in the salivary glands of *P. argentipes *(Figure 1A) and *P. perniciosus *(Figure 1B).

This family of proteins belong to the CAP family (CRISP, Ag5,PR-1) of proteins [37,35]. A remarkable feature of this family is the large number of cysteine residues, particularly at the carboxy-terminal region. X-ray structure of Na-AS-2, a member of this family from the human hookworm *Necator americanus*, was recently reported [38] and showed structural similarities to chemokines. Thus, it is possible that this type of protein in sandflies or other insects may bind cytokines with potential effects on the host immune response.

**Figure 8:**
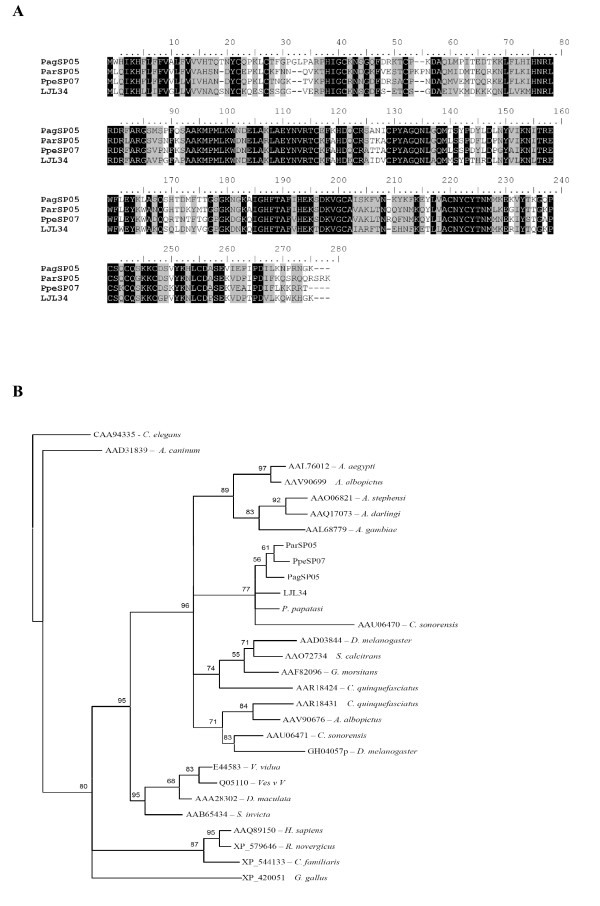
**Analysis of antigen 5-related proteins**. (A) Multiple sequence alignment of antigen 5 related proteins from the salivary glands of *Phlebotomus argentipes *(Pag), *Phlebotomus ariasi *(Par), *Phlebotomus perniciosus *(Ppe) *and Lutzomyia longipalpis *(LJ). Sequences were aligned using ClustalX and manually refined using BioEdit sequence-editing software. (B) Phylogenetic tree analysis of antigen 5-related protein from sandflies and other organisms, including *A. Aegypti, A. Albopictus, A. stephensi, A. darlingi, A. gambiae, C. sonorensis, D. melanogaster, S. calcitrans, G. morsitans, C. quinquefasciatus, V. vidua, D. maculata, S. invicta, H. sapiens, R. novergicus, C. familiaris *and *G. gallus*. Phylogenetic analysis was conducted on protein alignments using Tree Puzzle version 5.2 by maximum likelihood using quartet puzzling, automatically estimating internal branch node support (1000 replications).

Multiple alignments of the antigen-5 protein from the sandflies indicated a 49% identity and 80% similarity (Figure 8A) with fourteen conserved cysteines. Phylogenetic analysis identified unique clades containing Hymenoptera, Culicidae, sandflies and mammals (Figure 8B). The only other organism included in the sandfly clade was the biting midge *Culicoides sonorensis*.

#### 33-kDa protein family

**Figure 9:**
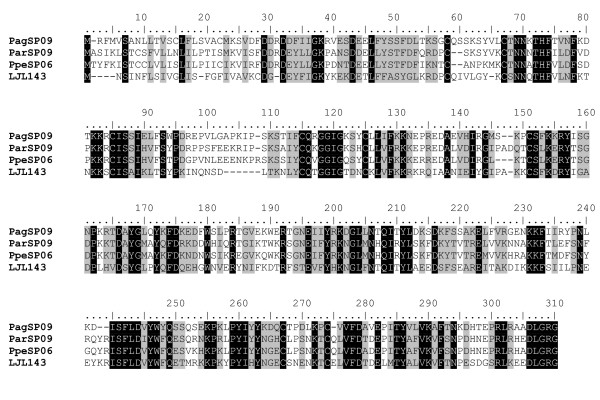
**Multiple sequence analysis of the 33-kDa protein family found on the salivary glands of sandflies**. *Phlebotomus *and *Lutzomyia *salivary proteins were used for this comparison. Sequences were aligned using ClustalX and manually refined using BioEdit sequence-editing software.

The 33-kDa protein family does not appear to be related to any other known proteins found in GenBank. *Phlebotomus ariasi *and *P. perniciosus*, 33-kDa proteins, are more closely related to each other than to *P. argentipes*, whereas the 33-kDa protein from *L. longipalpis *is distant to the three *Phlebotomus *species (data not shown). In general, all four sequences are somewhat similar with only 34% shared amino acids (Figure 9). Without further investigation, the function of this protein is unknown.

#### Endonuclease family of proteins

**Figure 10:**
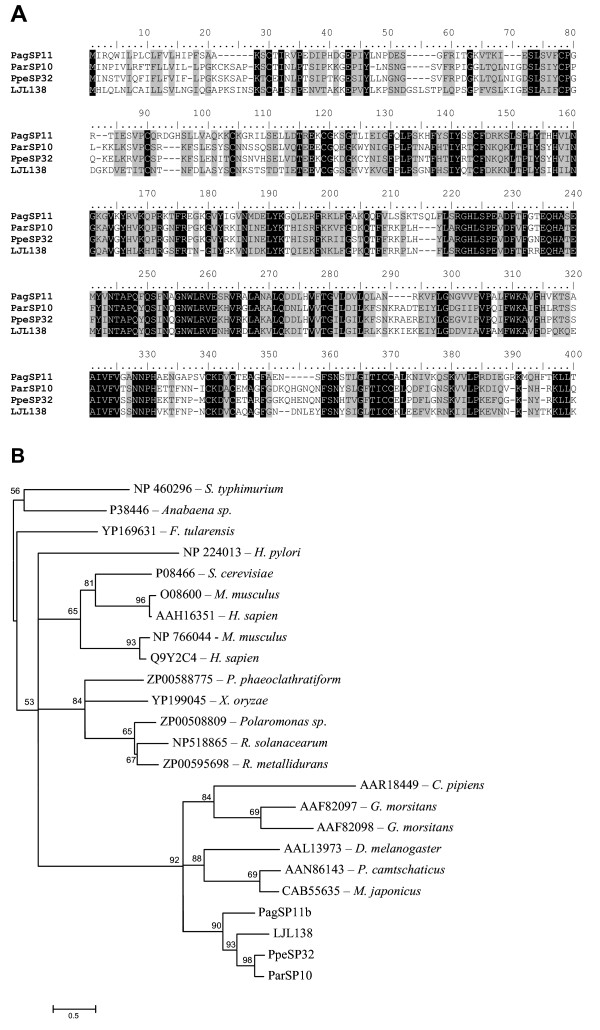
**Analysis of the endonuclease family of proteins**. (A) Multiple sequence analysis of salivary endonucleases from *Phlebotomus *and *Lutzomyia *sandflies. Sequences were aligned using ClustalX and manually refined using BioEdit sequence-editing software. (B) Phylogenetic tree analysis of endonucleases from sandflies and other organisms, *including S. thypimurium, Anabaena sp., F. tularensis, H. pylori, S. serevisiae, M. musculus, H. sapiens, P. phaeoclathratiform, X. oryzar, Polaromonas sp., R. solanacearum, R. metallidurans, C. pippiens, G. morsitans, D. melanogaster, P. cantschaticus *and *M. japonicus*. Phylogenetic analysis was conducted on protein alignments using Tree Puzzle version 5.2 by maximum likelihood using quartet puzzling, automatically estimating internal branch node support (1000 replications).

Transcripts coding for an endonuclease-like protein were found in *P. argentipes *(PagSP11) and *P. perniciosus *(PpeSP32) salivary gland cDNA libraries. These transcripts code for a protein of approximately 40 kDa with similarities to non-specific endonucleases from the sandfly *L. longipalpis *[33], tse tse fly *Glossina morsitans *[39], and the mosquito *Culex pipiens quinquefasciatus *[40]. This transcript does not have a direct match with the NUC Smart motif, which is indicative DNA/RNA non-specific endonucleases and phosphodiesterases, but it does have a high homology with other known endonucleases from other arthropods such as *D. melanogaster *(AAL13973) as well as non-insect arthropods such as *P. camtschaticus *(king crab) (AAN86143) and *M. japonicus *(prawn) (ACB55635) (Figure 10B).

Endonuclease proteins were found in all sandflies studied. The multiple alignment of sandfly endonuclease showed various regions of identity among amino acids, and many regions of conserved amino acids, even when comparing endonucleases from different sandfly genera (Figure 10A). Phylogenetic analysis indicated that the sandfly endonucleases clustered (92% bootstrap support) with other arthropods, including two non-insect arthropods (*Paralithode camtschaticus *and *Marsupenaeus japonicus*) (Figure 10B), however, sandfly endonucleases formed a distinct clade within the arthropod cluster (90% bootstrap support). Additionally, these endonucleases were clearly distant from other endonucleases. This suggests that this endonuclease may represent a common antigen for different sandflies and that the distant relationship to other endonucleases may avoid potential cross reactivity with non-insect organisms. Since this type of enzyme can cleave double- and single-stranded DNA, the role of this protein in the saliva of sandflies should be investigated further.

#### PpSP32-like protein

**Figure 11:**
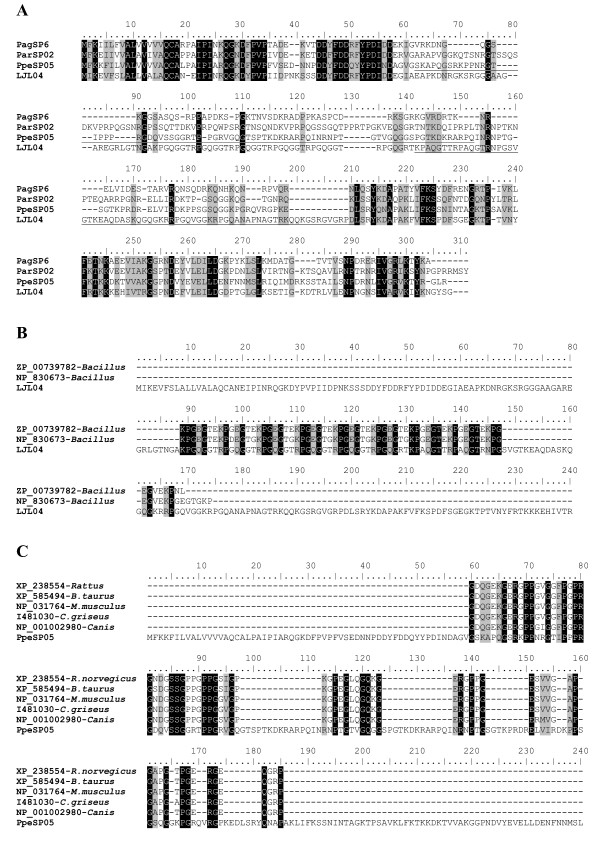
**Analysis of the PpSP32 protein family**. (A) Multiple sequence analysis of PpSP32 like proteins from the salivary glands of *Phlebotomus *and *Lutzomyia *sandflies. (B) Multiple sequence alignment of *Lutzomyia longipalpis *salivary protein LJL04 and collagen adhesion proteins from *B. thruingiensis *and *B. cereus*. (C) Multiple sequence alignment of Phlebotomus perniciosus PpeSP05 and type VII collagen proteins from *Canis familiairis*, *Mus musculus*, *Rattus norvegicus*, Chinese hamster, and *Bos Taurus*. Sequences were aligned using ClustalX and manually refined using BioEdit sequence-editing software.

The PpSP32-like family of proteins is similar to the 32.4-kDa protein first identified in *P. papatasi *salivary glands [14]. We found only one cluster (PagSP06) coding for this protein in the *P. argentipes *cDNA library. BLAST analysis of PagSP06 identified significant homology to *L. longipalpis *and *P. papatasi *PpSP32-like proteins. The *P. perniciosus *cDNA library contained only one cluster (PpeSP05) sharing identity with the PpSP32-like protein. Interestingly, upon BLAST analysis the PpSP32-like protein from *P. perniciosus *was found to be highly homologous to a Type VII collagen protein from *Canis familiaris *(E = 10^-5^) as well as a collagen from *Mus musculus*, *Rattus norvegicus*, Chinese hamster, and *Bos taurus*. This homology was found along approximately 74 amino acids (36% identity) and was dominated by conserved glycines and prolines (Figure 11).

The role of a collagen-like protein in sandfly salivary glands is unknown, yet intriguing. Because this protein may bind matrix protein, it is possible that this type of protein may form strong associations with basal matrix proteins. Within the *Phlebotomus *genera the relationship to collagen appears to be limited to PpSP32 kDa-like proteins from *P. pernicious*. *P. papatasi *and *P. ariasi *share only weak (non-significant) homology with Type VII collagen (E = 2.8 and 4.5, respectively) and *P. argentipes *did not match any collagen proteins upon multiple BLAST searches.

One explanation for the apparent homology between the four sandflies studied, yet lack of identity between collagen-related proteins in all sandflies, was revealed in the comparative analysis of the four sandflies. The majority of the homology between the four sandflies was found at the N- and carboxy terminus whereas the middle section of the protein appeared to be less conserved. The N-terminal and carboxy terminus has 43% and 23% identity between the four flies, respectively, whereas the midsection of the protein contains only 4% identity (Figure 11A). The region of homology between *P. perniciosus *and Type VII collagen was found in the non-homologous region of the protein (Figure 11B).

Additionally, BLAST analysis of *L. longipalpis *LJL04 (AAS16906) revealed a significant homology to collagen adhesion proteins from *B. thuringiensis *(ZP_00739782) and *B. cereus *(NP_830673) (Figure 11C). Again, the region of homology was found in the divergent section of the PpSP32-kDa protein. The most perplexing aspect of this family of proteins is that, although highly conserved among the four sandflies studied here, the N-terminal and carboxy regions of the protein have no homology to proteins of known function.

### Perspectives on the evolution of hematophagy in sandflies

The presence of a specific gene in distantly related species could indicate a true orthologous relationship (i.e., the presence of the gene in the ancestral species) or acquisition of the gene by one of the species by horizontal gene transfer. While horizontal gene transfer is a common event in prokaryotes its provenance in eukaryotes is not well established and the general consensus is that its occurrence is rare, if present at all. In the case of sandflies this would imply that proteins present in evolutionary distant species (insects or other metazoans) were present in the ancestral sandfly. As such, two general trends can be observed for the sandfly protein families found in their transcriptomes. Firstly, those proteins that occur throughout the metazoans or insecta tend to be found as single members in all sandfly genera with no extensive gene duplication events occurring in sandflies. These proteins are generally well conserved and possibly share the same or a very similar function to those found in the main family. These proteins include the apyrase, antigen 5 and endonuclease families and probably consist of the core repertoire of the ancestral sandfly proteins that develop during adaptation to hematophagous behavior. Although yellow-related proteins and D7 proteins are generally well conserved, we observed some gene expansion of these proteins in sandflies (Figures 3 and 7).

Alternatively, a number of protein families are limited to sandflies as a group or specific species and show low levels of similarity to family members found in other insects. Members of this group show high levels of divergence, more gene duplication events and were probably evolved specifically during adaptation to a blood-feeding lifestyle and specific host species. Proteins in this category include the PSP15 like, PSP32 like, 32 kDa, 39 kDa and 16.1 kDa protein families (Table 3) Within this group can also be placed the singletons, which are proteins limited exclusively to single species (Table 4). In the case of the 39 kDa and 16.1 kDa families (Table 3), it is possible that gene losses occurred among selected sandfly species or the sequences or transcripts were missed on this analysis due to low representation of these transcripts in the sandflies studied. In the case of the 2- and 5-kDa salivary proteins only found in *P. ariasi *and *P. perniciosus *(Table 4), these proteins may be specific for the subgenus *Larroussius*. We found many transcripts in the *P. argentipes *and *L. longipalpis *cDNA libraries coding for proteins of similar MW, however with no significant homology to these two proteins.

As expected, we found that salivary proteins from sand flies belonging to the same subgenus are more closely related to each other than proteins from different subgenus. We observed that proteins belonging to *P. ariasi *(subgenus *Larroussius*) in all phylogenetic tree analysis based on protein sequence were more closely related to *P. perniciosus *(subgenus *Larroussius*) than to *P. argentipes *salivary proteins (subgenus *Euphlebotomus*). These results are in agreement with previously studies using the small subunit nuclear ribosomal DNA [12].

### Can we use the salivary proteins common to these four sandflies as global antigens for a vector-based vaccine?

The large degree of divergence found in the majority of the most abundant sandfly salivary proteins suggests that a specific salivary protein may not be used as common vaccine target or as a common marker for sandfly exposure to different sandfly genera. This is supported by the recent findings by Rohousova et al [41], who compared antibody responses to salivary proteins from *P. papatasi*, *P. sergenti *and *L. longipalpis *and also demonstrated a lack of cross-reactivity between animals bitten by two different sandfly genera. This lack of cross-reactivity is possibly due to the low degree of similarities found in the *Lutzomyia *and *Phlebotomus *salivary proteins, as reported in the present work, and to the lack of recognition to specific molecules present exclusively in the different genera. We cannot exclude at this point potential cross-reactivity, or lack thereof, with components of the cellular immune response to sandfly salivary proteins. This area has been under-studied and should be evaluated experimentally. Although there is an overall low degree of identity between most salivary proteins across the genera, there are small regions of identities that may contain common T cell epitopes conserved between *Lutzomyia *and *Phlebotomus *salivary proteins. These small regions are observed in proteins such as D7, apyrases, yellow-related protein, antigen 5, a 33-kDa protein and endonucleases from these sandflies studied thus far.

In contrast, we found salivary proteins that have the potential to be a common vaccine target within the genus *Phlebotomus*. We identified three proteins that are highly conserved in different *Phlebotomus *species, the yellow-related protein, the apyrases and the antigen 5-related proteins. Additionally, these proteins have the potential to be markers of exposure for *Phlebotomus *sandflies in general. This is supported by observations by Rohousova et al [41] and Volf and Rohousova [42], which showed some cross-reactivity in animals to salivary proteins between different *Phlebotomus *species. These observations need to be expanded and evaluated experimentally for the potential cross-reactivity of these salivary proteins on specific cellular immune responses that may protect against *Leishmania *infection.

## Conclusion

Overall, this study led us to the identification of novel salivary proteins from two sandfly vectors of visceral leishmaniasis, *P. argentipes *and *P. perniciosus*, and the identification of the overall repertoire of secreted proteins present in their salivary glands. Additionally, this study allowed the discovery of the salivary proteins common among four different sandflies, from two different genera (*Lutzomyia *and *Phlebotomus*) and from two different subgenera (*Euphlebotomus *and *Larroussius*). This comparative study is giving us insight into the evolution of sandfly salivary proteins, their relationship and their molecular characteristics.

Moreover, this study is providing a better understanding of the overall sequence identity of sandfly salivary proteins across genus and species, while suggesting that a global vector-based vaccine may not be possible across different genera and that possibly genus- or species-specific salivary proteins may need to be used for this type of vaccine.

## Methods

### Sandfly rearing

Adult *Phlebotomus argentipes *(NIH colony) and *P. perniciosus *(kindly obtained by Dr. Michele Maroli, Italy) sandflies were kept with free access to a 20% solution of sucrose. Salivary glands from recently emerged and 1- to 2-day-old adult female flies were dissected and transferred to 10 or 20 ul 10 mM HEPES pH 7.0, 0.15 M NaCl in 1.5 ml polypropylene vials, usually in groups of 10 pairs of glands in 20 ul of HEPES saline. Salivary glands were kept at -75°C until needed.

### Salivary Gland cDNA Library

Salivary gland mRNA from both species was isolated from 40–50 salivary gland pairs, respectively, using the Micro-FastTrack mRNA isolation kit (Invitrogen, San Diego, CA). The PCR-based cDNA library was made using the SMART cDNA library construction kit (BD-Clontech, Palo Alto, CA), following the manufacturer's recommendation with some modifications [33]. The obtained cDNA libraries (large, medium and small sizes) were plated by infecting log phase XL1- blue cells (Clontech); insert size was determined with PCR using vector primers flanking the inserted cDNA and visualised on a 1.1 % agarose gel with ethidium bromide (1.5 ug/ml). Inserts were sequenced as previously described using a CEQ 2000XL DNA sequencing instrument (Beckman Coulter) [33].

### Bioinformatics

Detailed description of the bioinformatic treatment of the data appear in [19]. Briefly, primer and vector sequences were removed from raw sequences and sequences shorter than 50 nucleotides or containing more than 15% N were removed from further analysis. Sequences were compared to the GenBank non-redundant (nr) protein database using the standalone BlastX program [43] using a cut-off E-value of 1 × 10^-5^. Related sequences containing less than 5% N were clustered into rated groups based on 90% homology over a continuous stretch of 90 nucleotides using the CAP3 assembler program [44]. Sequences were then grouped into contigs and aligned. Contigs and singletons (contig containing only one sequence) were compared using the program BlastX, BlastN, or rpsBlast [43] to the non-redundant (nr) protein database of the National Center of Biological Information (NCBI), to the gene ontology database (GO) [45], to the Conserved Domains Database (CDD) that includes all Pfam [46], Smart [47,48] and COG protein domains in the NCBI [49].

Additionally, contigs were compared to a customised subset of the NCBI nucleotide database containing either mitochondrial (mit-pla) or rRNA (rrna) sequences. Identification of putative secreted proteins was conducted using the SignalP server [50]. The three-frame translation of each dataset was used to determine open reading frames (ORF). Only ORFs that started with a methionine and were longer than 40 amino acid (AA) residues were submitted to the SignalP server. The grouped and assembled sequences, BLAST results and signal peptide results were combined in an Excel spreadsheet and manually verified and annotated.

Sequence contigs containing signal peptides were selected from both species for further analysis and compared with secreted proteins from *L. longipalpis *[33] and *P. ariasi *[21]. Full-length secreted proteins from each sandfly library were compared using a stand alone version of BLAST [51] creating formatted protein databases of each sandfly library. Related groups of proteins from each sandfly species were combined into defined families of proteins.

To ensure the fidelity of the sandfly library comparative analysis, the original nucleotide sequence data files from each library (including secreted and non-secreted sequences) were combined and compared to known families of salivary gland proteins using BLAST analysis. Results of this analysis were compared to the original individual analysis.

Predictions of protein secondary structures were performed using the PHYRE prediction server [52].

### Phylogenetic analysis

The evolutionary relatedness of the protein families identified through the bioinformatics analysis was evaluated using phylogenetics. Consensus protein sequences of the identified protein families from each of the sandflies used in this analysis were compared with related sequences from non-visceral *Leishmania *sandfly vectors as well as non-sandfly species obtained from GenBank. Sequences were aligned using ClustalX [53] and manually refined using BioEdit sequence-editing software [54]. Alignments were analysed using ProtTest version 1.2.6 [55] to determine the best fit model of protein evolution for each particular alignment. Phylogenetic analysis was conducted on protein alignments using Tree Puzzle version 5.2 [56] incorporating the appropriate model of evolution defined by ProtTest. Tree Puzzle constructs phylogenetic trees by maximum likelihood using quartet puzzling, automatically estimating internal branch node support (1000 replications). Derived trees were visualised using TreeView [57].

### Full-length Sequencing of Selected cDNA Clones

An aliquot (4 μl) of the λ-phage containing the cDNA of interestwas amplified using the PT2F1 and PT2R1 primers, as described previously [33]. The PCR samples were cleaned using the multiscreen PCR 96-well filtration system (Millipore). Cleaned samples were sequenced first with PT2F3 primer (5'-TCT CGG GAA GCG CGC CAT TGT-3') and subsequently with custom primers.

### SDS-PAGE

For *P. argentipes *salivary glands, Tris-glycine gels (4–20%), 1 mm thick (Invitrogen), were used. Gels were run with Tris-glycine SDS buffer according to the manufacturer's instructions. To estimate the molecular weight of the samples, SeeBlue™ MW markers from Invitrogen were used. SGH were treated with equal parts of 2× SDS sample buffer (8% SDS in Tris-HCl buffer, 0.5 M, pH 6.8, 10% glycerol and 1% bromophenol blue dye). Each lane contained 20 pairs of homogenised *P. argentipes *salivary glands (20 ug protein). Protein were visualised with Coomassie blue stain. For aminoterminal sequencing of proteins, 20 pairs of homogenised salivary glands were electrophoresed and transferred to polyvinylidene difluoride (PVDF) membrane using 10 mM CAPS, pH 11.0, 10% methanol as the transfer buffer on a Blot-Module for the XCell II Mini-Cell (Invitrogen). The membrane was stained with Coomassie blue without acetic acid. Stained bands were cut from the PVDF membrane and subjected to Edman degradation using a Procise sequencer (Perkin-Elmer Corp.). Similar procedure was used to perform the amino-terminal sequence of *P. perniciosus *with the exception that 10% polyacylamide NuPAGE Bis-Tris (Invitrogen) was used for protein separation.

To locate the cDNA sequence cluster that corresponds to the amino acid sequence obtained by Edman degradation, we used a search program that compared the amino acid sequences against the three possible protein translations of each cDNA sequence obtained in the DNA sequencing project [22].

## List of abbreviations

COG, cluster of orthologous groups; OBP, odurant-binding protein; LSE, lineage-specific expansion; PLA2, phospholipase A2, MRJP, major royal jelly proteins; MW, molecular weight; PVDF, polyvinylidene difluoride;.

## Authors' contributions

JMA carried out the bioinformatics analysis, sequence alignment, phylogenetic analysis, helped in the design and coordination of the study and in drafting the manuscript. FO carried out multiple sequence alignments, phylogenetic analysis, helped in the coordination of the study and in drafting the manuscript. SK participated in conception of the study and sandfly rearing and revising the draft. BJM carried out phylogenetic analysis, sequence comparison and drafting part of the manuscript. DR carried out the proteomic analysis of sandfly salivary glands and drafting the Methods section of the manuscript. AES carried out sequencing and analysis of the *P. argentipes *transcripts and also drafting the Methods section. PL carried out the rearing of the *P. perniciosus *and *P. argentipes *colonies, provided salivary glands for the study and drafted part of the manuscript. MG carried out the Edman degradation and its analysis of all the proteins from this study. VMP carried out the sequence of the transcripts from the two cDNA libraries. JGV conceived the study, participated in its design and coordination, and drafting of the manuscript.

## References

[B1] Guerin PJ, Olliaro P, Sundar S, Boelaert M, Croft SL, Desjeux P, Wasunna WK, Bryceson AD (2002). Visceral leishmaniasis: current status of control, diagnosis, and treatment, and a proposed research and development agenda. The Lancet.

[B2] Ribeiro JMC, Francischetti IM (2003). Role of arthropod saliva in blood feeding: sialome and post-sialome perspectives. Annu Rev Entomol.

[B3] Belkaid Y, Kamhawi S, Modi G, Valenzuela JG, Noben-Trauth N, Rowton E, Ribeiro JMC, Sacks DL (1998). Development of a natural model of cutaneous leishmaniasis: powerful effects of vector saliva and saliva preëxposure on the long-term outcome of *Leishmania major *infection in the mouse ear dermis. J Exp Med.

[B4] Kamhawi S, Belkaid Y, Modi G, Rowton E, Sacks D (2000). Protection against cutaneous leishmaniasis resulting from bites of uninfected sand flies. Science.

[B5] Titus RG, Ribeiro JM (1988). Salivary gland lysates from the sand fly *Lutzomyia longipalpis *enhance Leishmania infectivity. Science.

[B6] Morris RV, Shoemaker CB, David JR, Lanzaro GC, Titus RG (2001). Sandfly maxadilan exacerbates infection with *Leishmania major *and vaccinating against it protects against *L. major *infection. J Immunol.

[B7] Valenzuela JG, Belkaid Y, Rowton E, Ribeiro JM (2001). The salivary apyrase of the blood-sucking sand fly Phlebotomus papatasi belongs to the novel Cimex family of apyrases. J Exp Biol.

[B8] Milleron RS, Mutebi JP, Valle S, Montoya A, Yin H, Soong L, Lanzaro GC (2004). Antigenic diversity in maxadilan, a salivary protein from the sand fly vector of American visceral leishmaniasis. Am J Trop Med Hyg.

[B9] Elnaiem DE, Meneses C, Slotman M, Lanzaro GC (2005). Genetic variation in the sand fly salivary protein, SP-15, a potential vaccine candidate against Leishmania major. Insect Mol Biol.

[B10] Deissler H, Lottspeich F, Rajewsky MF (1995). Affinity purification and cDNA cloning of rat neural differentiation and tumor cell surface antigen gp130RB13-6 reveals relationship to human and murine PC-1. J Biol Chem.

[B11] Rossetto O, Rigoni M, Montecucco C (2004). Different mechanism of blockade of neuroexocytosis by presynaptic neurotoxins. Toxicol Lett.

[B12] Aransay AM, Scoulica E, Tselentis Y, Ready PD (2000). Phylogenetic relationships of phlebotomine sandflies inferred from small subunit nuclear ribosomal DNA. Insect Mol Biol.

[B13] Charlab R, Valenzuela JG, Rowton ED, Ribeiro JM (1999). Toward an understanding of the biochemical and pharmacological complexity of the saliva of a hematophagous sand fly *Lutzomyia longipalpis*. Proc Natl Acad Sci USA.

[B14] Valenzuela JG, Belkaid Y, Garfield MK, Mendez S, Kamhawi S, Rowton ED, Sacks DL, Ribeiro JM (2001). Toward a defined anti-Leishmania vaccine targeting vector antigens: characterization of a protective salivary protein. J Exp Med.

[B15] Galindo K, Smith DP (2001). A large family of divergent Drosophila odorant-binding proteins expressed in gustatory and olfactory sensilla. Genetics.

[B16] Calvo E, Mans BJ, Andersen JF, Ribeiro JMC (2005). Function and evolution of a mosquito salivary protein family. J Biol Chem.

[B17] Arca B, Lombardo F, de Lara Capurro M, della Torre A, Dimopoulos G, James AA, Coluzzi M (1999). Trapping cDNAs encoding secreted proteins from the salivary glands of the malaria vector *Anopheles gambiae*. Proc Natl Acad Sci USA.

[B18] James AA, Blackmer K, Marinotti O, Ghosn CR, Racioppi JV (1991). Isolation and characterization of the gene expressing the major salivary gland protein of the female mosquito, *Aedes aegypti*. Mol Biochem Parasitol.

[B19] Valenzuela JG, Francischetti IM, Pham VM, Garfield MK, Mather TN, Ribeiro JM (2002). Exploring the sialome of the tick *Ixodes scapularis*. J Exp Biol.

[B20] Valenzuela JG, Pham VM, Garfield MK, Francischetti IM, Ribeiro JM (2002). Toward a description of the sialome of the adult female mosquito *Aedes aegypti*. Insect Biochem Mol Biol.

[B21] Oliveira F, Kamhawi S, Seitz AE, Pham VM, Guigal PM, Fischer L, Ward J, Valenzuela JG (2005). From transcriptome to immunome: Identification of DTH inducing proteins from a Phlebotomus ariasi salivary gland cDNA library. Vaccine.

[B22] Valenzuela JG, Charlab R, Gonzalez EC, de Miranda-Santos IK, Marinotti O, Francischetti IM, Ribeiro JM (2002). The D7 family of salivary proteins in blood sucking diptera. Insect Mol Biol.

[B23] Isawa H, Yuda M, Orito Y, Chinzei Y (2002). Mosquito salivary protein inhibits activation of the plasma contact system by binding to factor XII and high molecular weight kininogen. J Biol Chem.

[B24] Valenzuela JG, Ribeiro JM (1998). Purification and cloning of the salivary nitrophorin from the hemipteran *Cimex lectularius*. J Exp Biol.

[B25] Smith TM, Hicks-Berger CA, Kim S, Kirley TL (2002). Cloning, expression, and characterization of a soluble calcium-activated nucleotidase, a human enzyme belonging to a new family of extracellular nucleotidases. Arch Biochem Biophys.

[B26] Murphy DM, Ivanenkov VV, Kirley TL (2003). Bacterial expression and characterization of a novel, soluble, calcium-binding, and calcium-activated human nucleotidase. Biochemistry.

[B27] Grimaldi D, Engel MS (2005). Evolution of the insects. Evolution of Ectoparasites and Blood Feeders of Vertebrates.

[B28] Champagne DE, Smartt CT, Ribeiro JMC, James AA (1995). The salivary gland-specific apyrase of the mosquito Aedes aegypti is a member of the 5'-nucleotidase family. Proc Natl Acad Sci USA.

[B29] Sarkis JJ, Guimaraes JA, Ribeiro JMC (1986). Salivary apyrase of *Rhodnius prolixus*. Kinetics and purification. Biochem J.

[B30] Dai J, Liu J, Deng Y, Smith TM, Lu M (2004). Structure and protein design of a human platelet function inhibitor. Cell.

[B31] Geyer PK, Spana C, Corces VG (1986). On the molecular mechanism of gypsy-induced mutations at the yellow locus of *Drosophila melanogaster*. EMBO J.

[B32] Johnson JK, Li J, Christensen BM (2001). Cloning and characterization of a dopachrome conversion enzyme from the yellow fever mosquito, *Aedes aegypti*. Insect Biochem Mol Biol.

[B33] Valenzuela JG, Garfield M, Rowton ED, Pham VM (2004). Identification of the most abundant secreted proteins from the salivary glands of the sand fly *Lutzomyia longipalpis*, vector of *Leishmania chagasi*. J Exp Biol.

[B34] Gomes RB, Brodskyn C, de Oliveira CI, Costa J, Miranda JC, Caldas A, Valenzuela JG, Barral-Netto M, Barral A (2002). Seroconversion against *Lutzomyia longipalpis *saliva concurrent with the development of anti-*Leishmania chagasi *delayed-type hypersensitivity. J Infect Dis.

[B35] Lu G, Villalba M, Coscia MR, Hoffman DR, King TP (1993). Sequence analysis and antigenic cross-reactivity of a venom allergen, antigen 5, from hornets, wasps, and yellow jackets. J Immunol.

[B36] Francischetti IM, Valenzuela JG, Pham VM, Garfield MK, Ribeiro JM (2002). Toward a catalog for the transcripts and proteins (sialome) from the salivary gland of the malaria vector *Anopheles gambiae*. J Exp Biol.

[B37] Fang KS, Vitale M, Fehlner P, King TP (1988). cDNA cloning and primary structure of a white-face hornet venom allergen, antigen 5. Proc Natl Acad Sci USA.

[B38] Asojo OA, Goud G, Dhar K, Loukas A, Zhan B, Deumic V, Liu S, Borgstahl GE, Hotez PJ (2005). X-ray structure of Na-ASP-2, a pathogenesis-related-1 protein from the nematode parasite, *Necator americanus*, and a vaccine antigen for human hookworm infection. J Mol Biol.

[B39] Li S, Kwon J, Aksoy S (2001). Characterization of genes expressed in the salivary glands of the tsetse fly, *Glossina morsitans morsitans*. Insect Mol Biol.

[B40] Ribeiro JM, Charlab R, Pham VM, Garfield M, Valenzuela JG (2004). An insight into the salivary transcriptome and proteome of the adult female mosquito *Culex pipiens quinquefasciatus*. Insect Biochem Mol Biol.

[B41] Rohousova I, Ozensoy S, Ozbel Y, Volf P (2005). Detection of species-specific antibody response of humans and mice bitten by sand flies. Parasitology.

[B42] Volf P, Rohousova I (2001). Species-specific antigens in salivary glands of phlebotomine sandflies. Parasitology.

[B43] Altschul SF, Madden TL, Schaffer AA, Zhang J, Zhang Z, Miller W, Lipman DJ (1997). Gapped BLAST and PSI-BLAST: a new generation of protein database search programs. Nucleic Acids Res.

[B44] Huang M, Madan A (1999). CAP3: A DNA sequence assembly program. Genome Research.

[B45] Ashburner M, Ball CA, Blake JA, Botstein D, Butler H, Cherry JM, Davis AP, Dolinski K, Dwight SS, Eppig JT, Harris MA, Hill DP, Issel-Tarver L, Kasarskis A, Lewis S, Matese JC, Richardson JE, Ringwald M, Rubin GM, Sherlock G (2000). Gene ontology: tool for the unification of biology. Nat Genet.

[B46] Bateman A, Birney E, Durbin R, Eddy SR, Howe KL, Sonnhammer EL (2000). The Pfam protein families database. Nucleic Acids Res.

[B47] Schultz J, Milpetz F, Bork P, Ponting CP (1998). SMART, a simple modular architecture research tool: identification of signaling domains. Proc Natl Acad Sci USA.

[B48] Schultz J, Copley RR, Doerks T, Ponting CP, Bork P (2000). SMART: a web-based tool for the study of genetically mobile domains. Nucleic Acids Res.

[B49] Marchler-Bauer A, Panchenko AR, Ariel N, Bryant SH (2002). Comparison of sequence and structure alignments for protein domains. Proteins.

[B50] Nielsen H, Engelbrecht J, Brunak S, von Heijne G (1997). A neural network method for identification of prokaryotic and eukaryotic signal peptides and prediction of their cleavage sites. Int J Neural Syst.

[B51] BLAST. ftp://ftp.ncbi.nih.gov/blast/executables/.

[B52] PHYRE. http://www.sbg.bio.ic.ac.uk/~phyre.

[B53] Thompson JD, Gibson TJ, Plewniak F, Jeanmougin F, Higgins DG (1997). The CLUSTAL-X windows interface: flexible strategies for multiple sequence alignment aided by quality analysis tools. Nucleic Acids Res.

[B54] Bioedit. http://www.mbio.ncsu.edu/BioEdit/page2.html.

[B55] Abascal F, Zardoya R, Posada D (2005). ProtTest: selection of best-fit models of protein evolution. Bioinformatics.

[B56] Schmidt HA, Strimmer K, Vingron M, von Haeseler A (2002). TREE-PUZZLE: maximum likelihood phylogenetic analysis using quartets and parallel computing. Bioinformatics.

[B57] Page RD (1996). TreeView: an application to display phylogenetic trees on personal computers. Comput Appl Biosci.

